# Nitrous oxide inhalation combined with lidocaine local anesthesia on the comfort of plastic surgery outpatient procedures: a randomized, controlled, single-blind trial

**DOI:** 10.3389/fmed.2025.1683066

**Published:** 2025-10-29

**Authors:** Yidan Su, Jiefeng Zou, Jingwen Guo, Yan Qi, Yonghua Li, Bing Nie, Ziying Dong, Wenjun Zhang

**Affiliations:** ^1^Department of Plastic and Aesthetic Surgery, Qingdao Hospital, University of Health and Rehabilitation Sciences (Qingdao Municipal Hospital), Qingdao, China; ^2^Department of Burn and Plastic Surgery, Shanghai Changzheng Hospital, Naval Medical University, Shanghai, China; ^3^Department of Anesthesiology, Shanghai Changzheng Hospital, Naval Medical University, Shanghai, China; ^4^Department of Medical Cosmetology, Shanghai Skin Disease Hospital, Skin Disease Hospital of Tongji University, Shanghai, China

**Keywords:** nitrous oxide, local anesthesia surgery, randomized controlled trial, analgesia, anti-anxiety

## Abstract

**Background:**

The use of infiltration injection of lidocaine with adrenaline in outpatient plastic surgeries has certain limitations, such as injection pain, increased blood pressure, and heart rate fluctuations. Nitrous oxide has good analgesic and anti-anxiety effects. However, its application in outpatient plastic surgery is relatively limited. This study aimed to investigate the advances following combined nitrous oxide inhalation and local anesthesia to provide a new anesthesia option for improving surgical comfort.

**Methods:**

This study adopted a randomized, single-blind grouping method. After the preoperative assessment, patients inhaled nitrous oxide (up to 50% concentration) or air through a nasal mask. Before and during surgery, the patients’ basic information and vital signs were recorded. Follow-up was performed three days after surgery.

**Results:**

A total of 82 patients were randomized, with 41 in each group (Group A: N₂O/O₂; Group B: Air/O₂). Group A showed significantly better outcomes across multiple measures: maximum pain score was lower (1.9 ± 0.7 vs. 3.1 ± 0.9; mean difference: -1.2, 95% CI: −1.6 to −0.8; *p* = 0.0039), hemodynamic parameters were more stable, and anxiety reduction was greater (mean difference in BSTAI change: -0.6, 95% CI: −1.1 to −0.1; *p* = 0.018) compared to Group B.

**Conclusion:**

This combined anesthesia method significantly reduced the pain experienced during surgery, stabilized blood pressure and heart rate, reduced anxiety, and improved surgical comfort in outpatient plastic surgery patients.

**Clinical trial registration:**

Clinicaltrials.gov, identifier ChiCTR2400080612.

## 1 Background

Lidocaine with adrenaline is commonly used for local anesthesia at the surgical site and is characterized by a short anesthesia duration, limited local anesthetic dosage, and patient consciousness during surgery. Various factors may lead to dissatisfaction with the effects of surgical anesthesia in both doctors and patients, affecting intraoperative procedures and postoperative recovery. First, pain due to local anesthetic injections is usually unavoidable. Second, pain experienced by patients may cause emotional tension, resulting in increased heart rate, elevated blood pressure, and increased bleeding (1). Finally, although adrenaline can reduce lidocaine absorption, and enhance the anesthetic effect, it can also increase blood pressure and heart rate as well as patient anxiety and discomfort (2, 3). In addition to the need for treating physical ailments, patients who visit plastic surgery clinics prioritize the comfort and experience of treatment, and simple local anesthesia is insufficient to meet the anesthesia requirements. Therefore, there is a need to develop better anesthetic methods to improve patient comfort and surgeon satisfaction.

Nitrous oxide (N_2_O) is an inhalational anesthetic used worldwide. N_2_O has a weak anesthetic effect, with a minimum alveolar concentration (MAC) of 104%, usually used for anesthesia induction or maintenance in combination with other anesthetics (4). Although N_2_O is a weak anesthetic, it has a potent analgesic effect, and its combined use can help reduce the dosage of other anesthetic drugs (5). Aside from effectively controlling pain, it can also minimize the psychological effects of local anesthetic surgery on patients, reducing the risk of adverse cardiovascular events caused by lidocaine and adrenaline-induced blood pressure and heart rate fluctuations. Owing to its minimal impact on patient vital signs and fast postoperative consciousness recovery, N_2_O has been widely used in fields such as oral surgery (6, 7), emergency departments (8), and obstetrics and gynecology (9, 10) but is less frequently used in outpatient plastic surgery.

This study aimed to compare patient anxiety, pain scores, blood pressure, heart rate, and other indicators during local anesthesia surgery under N_2_O and oxygen inhalation conditions, analyze the differences in these indicators, and investigate the analgesic effects and surgical satisfaction.

## 2 Materials and methods

This study was registered with the Chinese Clinical Trial Registry (ChiCTR) under the registration number ChiCTR2400080612 and approved by the Ethics Committee of Qingdao Municipal Hospital (ethics number 2023-KY-068). Our work has been carried out in accordance with The Code of Ethics of the World Medical Association (Declaration of Helsinki) for experiments involving humans. Before the start of the trial, all participants were adequately informed and their signed consent forms were obtained.

### 2.1 Patient selection and inclusion criteria

The participants were patients who required local anesthesia for outpatient surgery with an age range of 18–65 years. The exclusion criteria were as follows: (1) allergy to medications used in this study; (2) patients undergoing facial surgery; (3) preoperative history of existing diseases or organ dysfunctions (including but not limited to cardiac, pulmonary, hepatic, renal, or neurological impairments); (4) long-term use of analgesics, sedatives, or psychiatric medications; (5) pregnant and lactating women; (6) patients unable to provide a comprehensive medical history.

### 2.2 Experimental procedure

In the calculation of the sample size for this study, based on the data from a preliminary survey, 41 patients were determined for each of Group A and Group B as the research sample size to ensure that the research results have high reliability and statistical significance. Randomization was performed using a computer-generated random sequence with fixed block sizes of 4 (2 patients allocated to Group A and 2 to Group B within each block). The randomization sequence was generated by an independent statistician and concealed in sealed, opaque envelopes that were opened only after patient enrollment. This ensured that the randomization team could not predict or influence the allocation of any given patient. After signing the informed consent form, patients underwent preoperative assessment scales, including the Brief State–Trait Anxiety Inventory (BSTAI) (11, 12). The randomization team checked, then opened the corresponding numbered randomization envelope, and informed the surgical team to proceed with the appropriate intervention. Group A (N_2_O/O_2_) received nitrous oxide/oxygen inhalation and Group B (Air/O_2_) received air/oxygen inhalation before undergoing the same local anesthesia and surgical procedure.

The patient lay supine on the operating table and was connected to electrocardiographic monitoring to assess the noninvasive blood pressure, heart rate, and pulse oxygen saturation (SpO_2_). After resting for 5 min, the anesthesiologist operated the inhalation device, administering N_2_O or air through a nasal mask to the patient. Based on literature evidence (13, 14) and device specifications, we determined the titration protocol to start at a concentration of 20% and increase by 5% every 5 min until reaching the maximum recommended concentration of 50%, which represents the optimal balance between efficacy and safety for outpatient procedures. Approximately 5 min after inhalation of 50% N_2_O or air, 2% lidocaine and 1:200,000 epinephrine was administered for local anesthesia, followed by the start of the surgical procedure. The assistant recorded the patient’s blood pressure, heart rate, and SpO_2_ at four time points: before inhalation, after anesthesia injection (measurements should be completed within ten minutes after local anesthesia injection), during surgery, and after inhalation cessation. The anesthesiologist closely monitored the patient’s condition throughout the procedure. In the event of adverse reactions such as nausea, vomiting, dizziness, or oxygen desaturation (SpO₂ < 92%), N₂O inhalation was immediately discontinued. Symptomatic relief measures were then initiated, including supplemental oxygen administration, positional adjustments, and pharmacological intervention if warranted. All adverse reactions were documented in detail, including their symptoms, time of onset, severity, and response to interventions.

Upon completion of surgery, inhalation was immediately stopped and pure oxygen was administered to both groups of patients for 10 min. After resting, postoperative assessments were completed. The primary outcome, maximal pain score during the surgical process, was assessed using the Visual Analog Scale (VAS). Secondary outcomes, also assessed using the VAS, included expected pain, average pain, and pain score at 5 min after surgery (9). Patient anxiety was re-evaluated using the BSTAI. We also briefly surveyed the operating surgeons regarding their satisfaction with and opinions on the combined anesthesia technique postoperatively.

Patients were followed up for one month post-surgery, primarily documenting safety indicators.

### 2.3 Statistical analysis

Data were analyzed using SPSS 22.0. The Shapiro–Wilk test was used to assess data normality. Categorical variables were analyzed using Fisher’s exact test, while within-group and between-group comparisons were performed using paired t-tests and independent t-tests, respectively. For repeated physiological measurements (blood pressure, heart rate, and SpO₂ at four timepoints), analysis of covariance (ANCOVA) was performed with baseline values (pre-inhalation) as covariates to compare group differences at subsequent timepoints. BSTAI score changes were analyzed by ANCOVA, adjusting for preoperative BSTAI scores and surgery duration. Subgroup analyses explored potential effect modifiers, including surgery duration and BSTAI score, with findings considered exploratory. The significance level was set at *α* = 0.05 (two-tailed). Data were verified by two independent personnel.

## 3 Results

### 3.1 Patient characteristics

**Figure 1:**
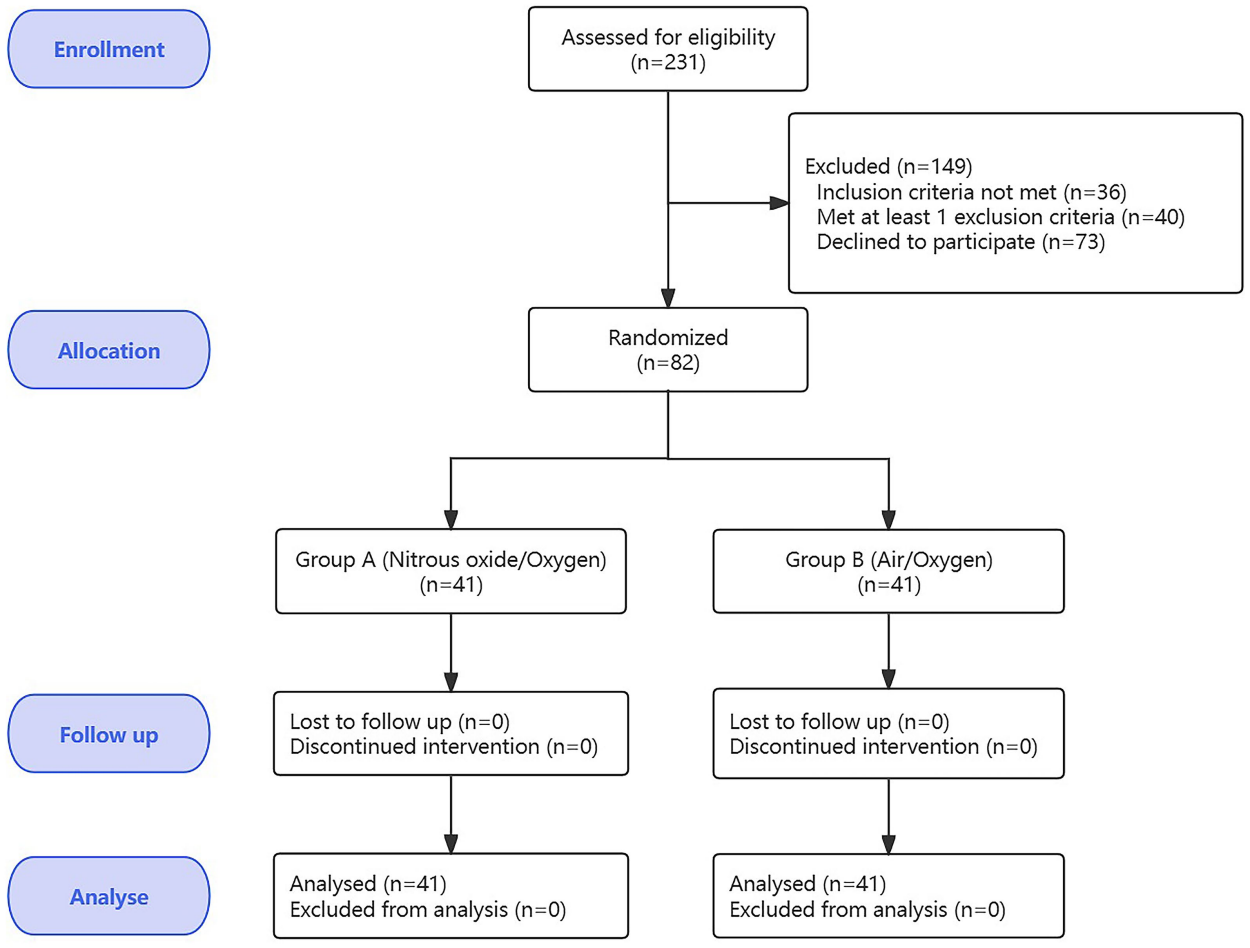
Flow diagram of participants.

From March 1, 2024, to March 1, 2025, we evaluated the eligibility of 231 patients, of whom, 36 did not meet the inclusion criteria, 40 met at least one exclusion criterion, and 73 declined to participate. As shown in Figure 1, 82 outpatients who underwent surgery completed the experiment, with 41 patients in Group A (N_2_O/O_2_) and 41 patients in Group B (Air/O_2_). The patient characteristics are summarized in Table 1. During the surgery and follow-up processes, no patients experienced adverse reactions such as dizziness, headache, nausea, vomiting, abdominal distension, hypotension (blood pressure <90/60 mmHg), arrhythmia, or agitation. All patients completed the 1-mouth follow-up.

**Table 1:** Patient characteristics.

Variable	Group A(N_2_O/O_2_) (*n* = 41)	Group B (Air/O_2_) (*n* = 41)	*P*-value
Age (years)	35.8 ± 4.6	32.9 ± 6.3	0.4264^&^
Gender			0.8253^#^
Female	22(53.7)	20 (48.8)	
Male	19 (46.3)	21 (51.2)	
Medical history			
Hypertension	5 (12.2)	7 (17.1)	0.7560^#^
Diabetes	8 (19.5)	5 (12.2)	0.5468^#^
Alcohol history^*^	3 (7.3)	6 (14.6)	0.4821^#^
Type of surgery			0.7915^#^
Excision of superficial tumor	12 (29.2)	15 (36.6)	
Wound debridement	5 (12.2)	4 (9.8)	
Circumcision	2 (4.9)	4 (9.8)	
Nail avulsion	3 (7.3)	1 (2.4)	
Incision and drainage	2 (4.9)	3 (7.3)	
Other	17 (41.5)	14 (34.1)	

Data are provided as mean ± SD or *n* (%). *History of heavy alcohol consumption, defined as drinking alcohol more than three times a week, consuming more than 60 g of pure alcohol on each occasion (equivalent to approximately 150 mL of spirits). ^&^Independent sample *t* test results. ^#^Fisher’s exact test results.

### 3.2 Visual analogue scale

After completion of the surgery, we collected the VAS scores from patients regarding maximum pain, expected pain, average pain, and pain 5 min after surgery (Table 2). In Group A, the maximum pain score was 1.9 ± 0.7, significantly lower than the expected pain score (mean difference: -1.1, 95% CI: −2.0 to −0.2, *p* = 0.0243), which was 3.0 ± 1.1. In Group B, the maximum pain score was 3.1 ± 0.9, and the expected pain score was 3.1 ± 1.5, with no significant difference between them. Results indicated that Group A had significantly lower maximum pain scores than Group B (mean difference: -1.2, 95% CI: −1.6 to −0.8, *p* = 0.0039). Approximately 39.0% (*n* = 16) of the patients in Group B reported that their maximum pain occurred during the administration of local anesthesia, whereas approximately 21.9% (*n* = 9) reported maximum pain during the surgical procedure. In Group A, approximately 43.9% (*n* = 18) of the patients reported that their maximum pain occurred during the surgical procedure. There were no significant differences in average pain, expected pain, and pain at 5 min after surgery within or between the two groups.

**Table 2:** Statistical analysis of visual analog scale scores.

Variable	Group A (N_2_O/O_2_) (*n* = 41)	Group B (Air/O_2_) (*n* = 41)	Mean difference (95% CI)	*P*-value
Maximum pain score	1.9 ± 0.7	3.1 ± 0.9	−1.2 (−1.6, -0.8)	**0.0039**
Expected pain score	3.0 ± 1.1	3.1 ± 1.5	−0.1 (−0.6, 0.4)	0.8664
Average pain score	1.8 ± 0.8	2.0 ± 0.7	−0.2 (−0.8, 0.5)	0.5480
Pain score at 5 min after surgery	1.3 ± 0.5	1.2 ± 0.4	0.1 (−0.5, 0.3)	0.6278

Data are provided as mean ± SD. *p*-values are from an independent sample *t*-test. Bold values indicate statistically significant results (*p* < 0.05).

### 3.3 Changes in blood pressure, heart rate, and SpO2

During surgery, we collected blood pressure (mean arterial pressure [MAP]), heart rate, and SpO_2_ values at four time points: before inhalation, after anesthesia injection, during surgery, and after inhalation cessation. The ANCOVA was performed with baseline values (before inhalation) as covariates. As shown in Table 3, the results revealed that Group A exhibited significantly lower MAP values compared to Group B after anesthesia injection (mean difference: -10.6, 95% CI: −15.1 to −6.1, *p* = 0.0006). Similarly, heart rate in Group A was significantly lower than in Group B after anesthesia injection (mean difference: -13.3, 95% CI: −19.1 to −7.5, *p* = 0.0035). No significant differences were observed in SpO_2_ between the two groups at any timepoint (all *p* > 0.05). The ANCOVA analysis adjusted for baseline values demonstrated that the differences in MAP and heart rate between the two groups remained statistically significant after accounting for initial measurements.

**Table 3:** Analysis of covariance (ANCOVA) for vital signs.

Variable	Timepoint*	Group A (N₂O/O₂)	Group B (Air/O₂)	Mean difference (95% CI)	*P*-value
MAP (mmHg)	1	98.1 ± 9.4	97.7 ± 11.3	0.4 (−3.2, 4.0)	0.8561
	2	96.9 ± 9.1	107.5 ± 12.4	−10.6 (−15.1, −6.1)	**0.0006**
	3	97.7 ± 8.2	101.1 ± 11.2	−3.4 (−7.2, 0.4)	0.0824
	4	97.2 ± 12.9	96.2 ± 9.5	1.0 (−3.5, 5.5)	0.4396
Heart rate (BPM)	1	75.8 ± 11.3	72.1 ± 11.9	3.7 (−1.2, 8.6)	0.2762
	2	73.9 ± 13.6	87.2 ± 12.2	−13.3 (−19.1, −7.5)	**0.0035**
	3	80.9 ± 14.9	79.0 ± 16.1	1.9 (−4.7, 8.5)	0.6335
	4	76.7 ± 13.3	75.9 ± 14.5	0.8 (−5.4, 7.0)	0.4479
SpO₂ (%)	1	98.6 ± 0.8	98.7 ± 1.1	−0.1 (−0.5, 0.3)	0.7566
	2	98.5 ± 1.4	98.9 ± 1.1	−0.4 (−1.0, 0.2)	0.2765
	3	98.8 ± 1.2	98.2 ± 1.3	0.6 (−0.1, 1.3)	0.0827
	4	97.9 ± 1.3	98.4 ± 1.3	−0.5 (−1.6, −0.2)	0.0525

*The time point is abbreviated as 1: Before inhalation; 2: After anesthesia injection; 3: During surgery; 4: After inhalation cessation. Data are presented as mean ± SD. ANCOVA was performed with baseline values (Timepoint 1) as covariates. Bold values indicate statistically significant results (*p* < 0.05). MAP, mean arterial pressure; BPM, beats per minute; SpO_2_, pulse oxygen saturation.

### 3.4 Brief state–trait anxiety inventory score

The statistical analysis of BSTAI for each group was presented in Table 4. The result of ANCOVA demonstrated that after incorporated preoperative BSTAI scores as a covariate to adjust for initial anxiety levels, Group A showed a significantly greater reduction in BSTAI scores compared to Group B (mean difference: -0.6, 95% CI: −1.1 to −0.1; *p* = 0.018). Subgroup analyses further revealed that the anxiolytic effect of N₂O was more pronounced in patients with surgery duration ≥45 min (mean difference: -0.8, 95% CI: −1.4 to −0.2; *p* = 0.008) and in those with high preoperative anxiety (Preoperative BSTAI ≥9; mean difference: -0.7, 95% CI: −1.2 to −0.2; *p* = 0.006). These findings suggested that N₂O provides greater clinical benefits for patients undergoing prolonged procedures and those with elevated preoperative anxiety levels.

**Table 4:** Analysis of covariance (ANCOVA) for Brief State–Trait Anxiety Inventory (BSTAI) scores.

Variable	Group A (N₂O/O₂)	Group B (Air/O₂)	Mean difference (95% CI)	*P*-value
BSTAI change^*^	−1.4 ± 0.9	−0.8 ± 1.1	−0.6 (−1.1, −0.1)	**0.018**
Subgroup analysis				
- Surgery duration ≥45 min	−1.5 ± 1.0	−0.7 ± 1.2	−0.8 (−1.4, −0.2)	**0.008**
- Surgery duration <45 min	−1.2 ± 0.8	−0.9 ± 1.0	−0.3 (−0.8, 0.2)	0.210
- Preoperative BSTAI ≥9	−1.6 ± 0.8	−0.9 ± 1.0	−0.7 (−1.2, −0.2)	**0.006**
- Preoperative BSTAI <9	−1.1 ± 0.9	−0.7 ± 1.1	−0.4 (−0.9, 0.1)	0.120

*BSTAI Change was calculated as postoperative scores minus preoperative scores. Data are presented as mean ± SD. ANCOVA was performed with preoperative BSTAI scores and surgery duration as covariates. Bold values indicate statistically significant results (*p* < 0.05).

## 4 Discussion

Nitrous oxide was initially used as an anesthetic in dental surgery and was one of the earliest anesthetics used in human medicine. Owing to its limited general anesthetic effect, it is now often used in combination with other anesthetics (4). N₂O exhibits a favorable safety profile in short-term administration, characterized by absence of respiratory tract irritation and minimal hepatotoxic or nephrotoxic effects. However, prolonged exposure to N₂O may inhibit methionine synthase activity, potentially resulting in hematological or neurological sequelae (15, 16). These risks are considered highly unlikely given the short duration of exposure such as those evaluated in the present study. The effects of N_2_O take only 30–40 s to occur after inhalation, with a pronounced analgesic effect and a weak anesthetic effect (5). Patients remain conscious after N_2_O inhalation, avoiding complications associated with general anesthesia, and postoperative recovery is rapid. In plastic surgery, patients seek treatment not only for medical conditions but also to enhance their appearance, leading to higher expectations for treatment comfort. N₂O inhalation addresses these needs by reducing discomfort, pain, and anxiety during procedures. To our knowledge, this is the first randomized, single-blind trial to investigate the combined effects of inhaled N₂O and local anesthesia on surgical analgesia and patient satisfaction in outpatient plastic surgery.

N_2_O has anesthetic and analgesic effects (17). The excellent analgesic effect of N_2_O has been confirmed by numerous studies and is widely used in clinical practice. For example, Singh et al. (9) on 140 patients undergoing abortion surgery confirmed that inhaled N_2_O effectively reduced surgical pain. Our study results also indicate that compared to the control group, inhaled N_2_O effectively reduced the maximum pain score experienced by patients, especially the pain caused by the injection of anesthetic drugs. In addition to its analgesic effect, N_2_O can reduce the increase in blood pressure and heart rate caused by adrenaline. For example, Wang et al. (18) found that N_2_O inhalation during endoscopic ultrasound-guided fine needle aspiration for digestive tract diseases resulted in stable blood pressure, heart rate, and blood oxygen saturation, with increased satisfaction among patients and doctors. The blood pressure stabilization function of N_2_O has also been confirmed in studies by researchers (19). This study found that the time point at which blood pressure and heart rate were most likely to fluctuate was after anesthetic injection, and the blood pressure and heart rate in the N_2_O inhalation group were significantly more stable than those in the control group. In our study, we found that the analgesic and blood pressure-stabilizing effects of N_2_O were mainly evident after anesthetic drug injection, while the advantages of N_2_O inhalation were masked after the local anesthetic took effect; after that point, there were no significant differences in various indicators between the experimental and control groups. Therefore, we suggest gradually reducing the concentration of N_2_O 15–20 min after local anesthetic injection to maintain inhalation at a lower concentration.

N_2_O also called “the laughing gas,” has a good anti-anxiety effect, which has been widely supported by previous studies, including research by Guimarães (20) and others, highlighting its potential in adjunctive anxiety therapy (21). In our study, the analysis of BSTAI scores demonstrated that N₂O combined with local anesthesia significantly reduced anxiety of patients, especially who with longer surgery duration and higher preoperative anxiety level. Future studies should expand the sample size and include more patients with complex surgical needs or elevated anxiety to further validate these results.

In our postoperative evaluations and follow-up processes, we also investigated the operating surgeons’ satisfaction with the administration of inhaled N_2_O. In practical terms, operating surgeons stated that the use of N_2_O inhalation adjunctive anesthesia could significantly reduce the dosage of local anesthetics. Furthermore, inhalation-assisted local anesthesia can increase patient compliance during surgical procedures such as changing or maintaining surgical positions. To minimize deviations caused by differences in N_2_O inhalation concentrations among patients, we uniformly set the maximum N_2_O concentration at 50%. However, in practical terms, surgeons have noted that for patients sensitive to N_2_O, setting higher inhalation concentrations could lead to increased speech and movement, potentially contaminating the surgical area. Therefore, in daily practice, we recommend selecting the inhalation concentration of N_2_O based on the patient’s tolerance level. In addition, preparations for inhalation requires longer preparation and additional staff, making it less suitable for short, minimally invasive procedures. Therefore, we believe that N_2_O combined with local anesthesia is more suitable for surgeries with, patients sensitive to pain, and longer surgical durations.

It is also important to acknowledge the environmental and occupational safety concerns associated with N₂O. As a greenhouse gas with a high global warming potential and long atmospheric lifetime, N₂O contributes to climate change when released into the atmosphere (22). Moreover, chronic occupational exposure to trace N₂O has been linked to a range of adverse health effects, ranging from hematological and neurological disorders to genetic and reproductive toxicities (23). These concerns have led to a decline in its routine use in operating theaters. Ensuring safety in short-duration outpatient plastic surgery requires robust exposure control measures, including N₂O scavenging systems, adequate ventilation, dedicated treatment rooms, proper staff training, and regular safety audits (24). Future implementation of N₂O in ambulatory settings should prioritize these integrated safety protocols to minimize environmental release and protect clinical staff.

The inclusion criteria in this study restricted the minimum age for N_2_O inhalation to 18 years to avoid adverse effects in children. In pediatric dentistry, the rapid and reversible nature of N_2_O makes it a valuable tool for managing mild to moderate anxiety in children. A study published by Wilson et al. (25) indicated that 61% of 1,758 surveyed dental practitioners used N_2_O in conjunction with other sedatives, and other studies (26–28) have also confirmed the efficacy and safety of N_2_O in children. In plastic surgery emergencies, many children with skin lacerations may be uncooperative due to crying, making it challenging to perform precise wound-closure procedures. For children with phimosis, administering anesthesia and maintaining proper positioning during surgery pose significant challenges. The results of this trial suggest that N_2_O inhalation combined with local anesthesia can effectively reduce the pain associated with anesthetic injections, decrease patient anxiety at appropriate concentrations, and enhance surgical compliance, thereby addressing common issues in pediatric outpatient surgery. We also aimed to investigate the efficacy and safety of N_2_O inhalation during pediatric surgery in subsequent studies.

## 5 Conclusion

This trial introduced the use of N_2_O inhalation in combination with local anesthesia in outpatient plastic surgery procedures and conducted surveys and statistical analyses of patient pain scores, vital signs, and anxiety ratings. These results indicated that N_2_O inhalation combined with local anesthesia decreased pain during outpatient plastic surgery, especially during local anesthetic injections, reduced intraoperative blood pressure and heart rate fluctuations, alleviated anxiety in surgical patients, and improved patient comfort. These findings support the use of this technique in minor outpatient procedures. Further investigation is warranted to establish its utility across a broader range of surgical scenarios.

## Data Availability

The original contributions presented in the study are included in the article/supplementary material, further inquiries can be directed to the corresponding author/s.
